# Insights from computational modeling on the potential hemodynamic effects of sinus rhythm versus atrial fibrillation

**DOI:** 10.3389/fcvm.2022.844275

**Published:** 2022-09-14

**Authors:** Matteo Anselmino, Stefania Scarsoglio, Luca Ridolfi, Gaetano Maria De Ferrari, Andrea Saglietto

**Affiliations:** ^1^Division of Cardiology, Department of Medical Sciences, “Città della Salute e della Scienza di Torino” Hospital, University of Turin, Turin, Italy; ^2^Department of Mechanical and Aerospace Engineering, Politecnico di Torino, Turin, Italy; ^3^Department of Environmental, Land, and Infrastructure Engineering, Politecnico di Torino, Turin, Italy

**Keywords:** atrial fibrillation, hemodynamics, beat-to-beat variability, systemic circulation, cerebral circulation, coronary circulation

## Abstract

Atrial fibrillation (AF) is the most common clinical tachyarrhythmia, posing a significant burden to patients, physicians, and healthcare systems worldwide. With the advent of more effective rhythm control strategies, such as AF catheter ablation, an early rhythm control strategy is progressively demonstrating its superiority not only in symptoms control but also in prognostic terms, over a standard strategy (rate control, with rhythm control reserved only to patients with refractory symptoms). This review summarizes the different impacts exerted by AF on heart mechanics and systemic circulation, as well as on cerebral and coronary vascular beds, providing computational modeling-based hemodynamic insights in favor of pursuing sinus rhythm maintenance in AF patients.

## Introduction

Atrial fibrillation (AF), the most common clinical tachyarrhythmia in adults, provokes a significant burden to patients, physicians, and healthcare systems worldwide. The Global Burden of Disease (GBD) Study estimates nearly 60 million prevalent AF cases in 2019 (nearly doubling the estimated prevalence in 1990) (1), with epidemiological projections foreseeing a further rise during the next decades (2). These alarming data urge the scientific community to attempt solving the clinical conundrum concerning the optimal management of this growing subset of patients.

The never-ending debate between those favoring sinus rhythm (SR) maintenance (*rhythm control*) over the simple treatment of symptoms related to high ventricular response AF (*rate control*) is yet not concluded. In the early 2000s, two randomized clinical trials (AFFIRM and RACE) showed, with respect to hard cardiovascular endpoints, that rate control was non-inferior to rhythm control. Subsequent data pooled from a large population of patients (3) reported similar results, and the AF-CHF study (4) showed that, even in the presence of heart failure, rhythm control did not result superior to rate control. However, these trials were published before the widespread adoption of AF catheter ablation, and thus, the rhythm control option mostly included patients on anti-arrhythmic drugs (AADs). In the last decade, instead, AF catheter ablation has emerged as the most effective tool to maintain long-term SR, both as second-line therapy after failed AADs or as first-line approach (5, 6), potentially modifying the rhythm versus rate control benefits. In fact, a *post-hoc* analysis of the AFFIRM trial showed that SR rhythm maintenance was independently associated with improved survival, hinting that rhythm control might be prognostic, in case SR is maintained avoiding AADs side effects (7). In the past few years, albeit AF catheter ablation missed to statistically demonstrate its superiority on hard cardiovascular endpoints over AAD-based rhythm control strategy in the intention-to-treat analysis of a large clinical trial (CABANA) (8), the per-protocol analysis of the aforementioned study (characterized by high crossover rates between study arms), as well as several observational studies (9–11), showed that AF catheter ablation reduced all-cause mortality, stroke, and hospitalization. The EAST-AFNET 4 trial (12), published in 2020, eventually demonstrated that an early rhythm control approach, within the first 12 months since the first diagnosis, both by AADs and AF catheter ablation, was associated with improved survival compared to a standard approach (e.g., rate control, with rhythm control reserved to those patients still symptomatic despite rate control medications).

Clinical data focusing on the hemodynamic effects of AF are lacking and somewhat controversial, likely due to the challenge of obtaining precise measures in the specific districts (e.g., cerebral circulation) and of eliminating the numerous potential clinical confounders that limit the attempt of investigating arrhythmia-specific effects. The present review, sustained by the mounting data toward the prognostic benefit of SR maintenance, summarizes modeling-based evidence on the detrimental hemodynamic effects of AF on different circulatory districts to thoroughly argument why restoring SR in AF patients should be pursued. In particular, we focused on the three main aspects that have captured the most attention in the last decade, namely, heart mechanics and systemic circulation, cerebral hemodynamics, and coronary circle.

**FIGURE 1:**
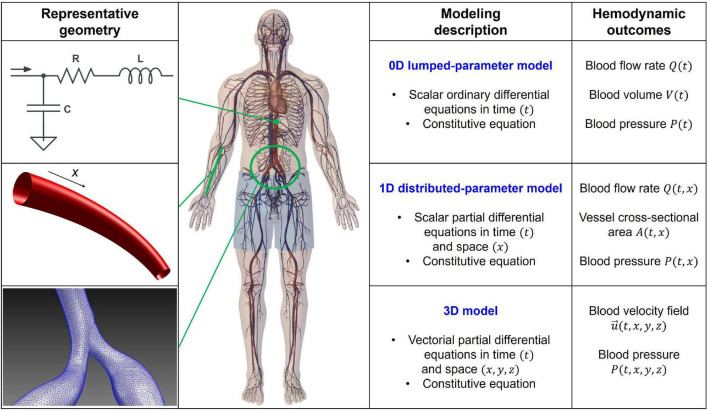
Scheme of the 0D, 1D, and 3D computational approaches: representative geometry, modeling description, and hemodynamic outcomes. RLC circuit accounts for dissipative (*R*, resistance), inertial (*L*, inertance), and elasticity (*C*, compliance) effects in the 0D model. Spatial coordinates (*x*, *y*, *z*) and time (*t*) are the independent variables, while volume (*V*), flow rate (*Q*), pressure (*P*), vessel cross-sectional area (*A*), and velocity field ($\vec{u}$=(*u, v, w*)) are the dependent variables and hemodynamic outcomes of the different models. Balance equations of mass and momentum are expressed in the differential form (0D: ordinary; 1D and 3D: partial), while constitutive equations rule the strain-stress relation between blood and wall vessel.

The efficiency of multiscale hemodynamic modeling for the description of the cardiovascular system has been widely recognized, and personalized computational hemodynamics is currently a reliable investigative tool in translational medicine (13–19). Figure 1 shows a schematic representation of the main computational approaches, ranging from zero-dimensional (0D) to three-dimensional (3D), together with their mathematical features and hemodynamic outcomes. By simplifying the spatial description, in the 0D lumped-parameter model, each cardiovascular region is modeled through a combination of electrical counterparts: the viscous/dissipative effects are taken into account by the resistances (R), the distensibility/contractility effects are described by the compliances/elastances (C/E), and the inertial effects are considered by the inertances (L), leading to the most general 3-elements (or RLC circuit) Windkessel model. Through the electric analog, the only independent variable is time (t), and the 0D lumped-parameter model is suitable to investigate the temporal evolution of each cardiovascular compartment in terms of pressure (P), flow rate (Q), and blood volume (V). The one-dimensional (1D) distributed-parameter model identifies the vessel axis (x) as the preferential spatial direction and assumes the following hypotheses: the blood is Newtonian, incompressible, and characterized by constant density and kinematic viscosity; effects of suspended particles are neglected; vessels are asymmetrical, tapered, longitudinally tethered, with impermeable walls and only subject to small and radial deformations; flow is laminar; and pressure is uniform across the section. *x* and *t* (time) are the independent variables, while hemodynamics is described by the vessel area (A), pressure (P), and flow rate (Q). The 1D model, composed of the continuity, momentum, and constitutive equations, is able to capture a higher level of geometrical and viscoelastic details, as well as wave propagation in the arterial tree. Eventually, in the 3D model, the blood flow is governed by the three-dimensional continuity and momentum Navier-Stokes equations, which are discretized over the computational domain and usually solved by finite element/volume methods. In so doing, the temporal evolution of the 3D flow velocity and pressure fields over the whole spatial domain are obtained, making 3D modeling particularly promising to investigate the local hemodynamics of complex vascular morphologies in terms of flow velocity and wall shear stress-related parameters. Even though hemodynamic modeling cannot substitute or deny *in vivo* clinical findings, modeling-based data provide important insights for clinicians and lay the basis for future dedicated clinical studies (20–23).

## Atrial fibrillation, heart mechanics, and systemic circulation

The AF induces several detrimental effects on systemic hemodynamics. The two main determinants of this noxious influence are (1) the fibrillatory atrial activation during AF that leads to ineffective atrial contraction and loss of atrial contribution to the ventricular filling (“atrial kick,” normally contributing up to 20–30% of the volume) (24); and (2) the irregularity of the ventricular activation during AF (irregular RR intervals), as elegantly demonstrated in a seminal study by Clarke (25), published more than 20 years ago. In this study, performed on 16 AF patients, compared to ventricular pacing at the same mean heart rate, during AF, the irregularity of RR intervals led to decreased cardiac output, increased pulmonary wedge pressure, and increased right atrial pressure.

However, despite these known effects, evidence on the global cardiovascular system response to AF remained scarce and controversial. Recently, a lumped-parameter AF modeling approach for the heart and systemic circulation was proposed (26), presenting the following potential advantages: first of all, AF’s hemodynamic impact can be assessed in standardized conditions, without the potential confounding effect of other comorbidities; second, the most relevant cardiac variables, as well as hemodynamic parameters, can be monitored contemporaneously. The initial computational analysis investigated the hemodynamic response during AF at a given mean ventricular rate (75 bpm) in an ideal healthy male young adult, meaning that the model is not patient-specific, but model parameters are calibrated to reproduce the hemodynamic response of a generic healthy subject with common anthropometric and physical features (e.g., weight: 75 kg, height: 175 cm, age: 25 years old, sex: male). In this setup, AF induced a reduction in cardiac output, stroke volume, and ejection fraction, as well as an increase in left ventricular end-diastolic pressure and volume, left atrial pressures and volumes, and pulmonary vein pressure. Overall, while the right chambers appeared to be less affected by the arrhythmia, left ventricle pressure-volume loops clearly indicated reduced cardiac efficiency during AF compared to SR. A focus on the fluid dynamics of heart valves (27) showed that during AF, both atrioventricular valves do not seem to worsen their performance, while the arterial valves’ efficiency is remarkably reduced.

A further step forward was to investigate the hemodynamic effects of different mean ventricular response rates. In fact, from a clinical standpoint, a clear heart rate target for AF patients, especially for those with the permanent form of arrhythmia, is lacking. A single randomized clinical trial, the Rate Control Efficacy in Permanent Atrial Fibrillation II (RACE II) (28), suggested that, in patients with permanent AF, lenient (targeting resting heart rate below 110 bpm) is not inferior to strict rate control (targeting resting heart rate below 80) both in terms of cardiovascular outcomes and quality of life. However, this clinical study presented several limitations, one out of all the modest difference in average heart rates achieved in the lenient and strict control groups (85 and 75 bpm, respectively) (29). Five different computational simulations, assuming resting conditions, with varying mean ventricular responses (50, 70, 90, 110, 130 bpm) were therefore performed (30). Interestingly, based on the lumped parameter model, slower ventricular responses during AF related to reduced left ventricular pressure increased stroke volume and ejection fraction, improved cardiac efficiency, and reduced oxygen consumption compared to higher ventricular rates. These results suggest that lowering ventricular rate during AF may partially blunt the detrimental hemodynamic impact exerted by the arrhythmia. Furthermore, an additional analysis was run to evaluate how the resting ventricular rate influenced the global cardiovascular systemic response to exercise with ongoing AF (31). Once again, the outcome of this exploration underlined that, in case of a slower basal ventricular response (70 bpm), compared to a higher one (100 bpm), the pulmonary venous pressure undergoes a dampened worsening (increase), and systemic blood pressure shows a more appropriate increase (as demanded by exertion).

The same computational framework was used to assess the hemodynamic impact of different left valvular heart diseases on systemic hemodynamics during AF (32). Several valvular pathologies (e.g., aortic stenosis, aortic regurgitation, mitral stenosis, and mitral regurgitation), with different grades of severity (i.e., mild, moderate, and severe), were simulated. Based on computational outcomes, regurgitant valvular diseases strongly affected AF hemodynamics (reduced cardiac output and systemic pressure, increased left ventricular volume, left atrial pressure, and pulmonary vein pressure), while aortic stenosis was the least impacting among the simulated valvular conditions. Given that AF is rarely an isolated pathology, in our opinion, these data provide a clear additional insight: if associated with comorbid conditions, such as valvular heart disease, AF may act as a further trigger toward hemodynamic decompensation.

More sophisticated multiscale approaches are able to grasp propagation and waveform alterations as well as regional variations of flow structure induced by AF. By comparing SR and AF at the same given mean ventricular rate (75 bpm), a multiscale 0D-1D modeling (33), which couples a 0D cardiac dynamics and a 1D description of the arterial tree, showed that the arterial system is not able to completely absorb the AF-induced variability. The sole heart rhythm variation promotes an alteration of the wave dynamics, which is amplified in the distal circulation, by modifying the interplay between forward and backward signals. The results suggest a possible vascular dysfunction due to prolonged exposure to irregular and extreme values. Recent 0D-3D multiscale studies (34), coupling 3D PC-MRI data for the aorta and a compact 0D model for the remaining circulation, showed that AF led to the modification of systemic blood perfusion and increase of the endothelial cell activation potential. Both these mechanisms can increase the risk of atherogenesis and thrombus formation in different regions from ascending to the thoracic aorta. Moreover, the concomitant presence of aortic aging further worsens flow circulation (35): in this case, AF exacerbates the vascular defects due to aging, which increases the possibility of cardiovascular diseases *per se*.

**FIGURE 2:**
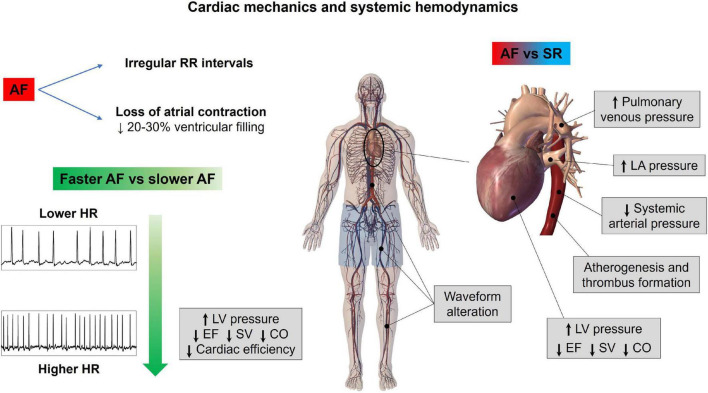
Atrial fibrillation effects on cardiac mechanics and systemic hemodynamics. AF, atrial fibrillation; CO, cardiac output; EF, ejection fraction; HR, heart rate; LA, left atrium; LV, left ventricle; SR, sinus rhythm; SV, stroke volume.

Notably, the aforementioned computational approaches focus on acute hemodynamic effects. Over longer time periods, the contractile impairment induced by fast and irregular ventricular rate (36) may induce arrhythmia-induced cardiomyopathy, and consequent detrimental systemic hemodynamic effects further magnify the hemodynamic alterations described. Figure 2 graphically summarizes the main concepts of this section.

## Atrial fibrillation and cerebral hemodynamics

Since at least three decades, it has been known that AF relates to a fivefold increased risk of stroke, compared to the general population (37). However, more recently, AF has shown to also relate to cognitive decline and dementia, independently from clinical cerebrovascular events (stroke or transient ischemic attack – TIA). A seminal work from Ott et al. (38), based on a cross-sectional analysis of a subgroup of patients in the Rotterdam Study, reported for the first time an independent association between AF and dementia. Thereafter, several prospective studies have been published, whose results have been recently integrated in a meta-analysis (39), demonstrating that AF is associated with a 28% increase in the risk of dementia compared to non-AF controls, net of the eventual intercurrent stroke/TIA during follow-up. However, the relative independent contribution of AF to dementia onset compared to other common dementia risk factors (e.g., age and hypertension) is to date not quantifiable. Another intriguing finding was that, when comparing the results to several previous analyses that assessed the risk of dementia without accounting for the possible occurrence of stroke/TIA during follow-up, the stroke-independent contribution to dementia seemed to be more prominent than the stroke-dependent one.

During the past years, several mechanisms have been proposed to explain the association between AF and cognitive decline (40). As reported by our group (41), AF patients present at cerebral magnetic resonance imaging (MRI) a significantly increased number of silent cerebral ischemia (SCIs) compared to a control group with a similar cardiovascular risk profile, and the burden of these lesions is proportional to the duration of the arrhythmia (paroxysmal to persistent). More recently, Conen (42) demonstrated that in 1,390 AF patients without previous cerebrovascular events, cerebral MRI showed the presence of different silent cerebral lesions in a significant number of patients, such as silent cerebral infarction in 30%, microbleeds in 22%, and white matter hyperintensities (WMHs) in 99% of the investigated population; the presence of these alterations, particularly silent infarctions and moderate-grade WMHs, was significantly associated with cognitive decline at multivariate analysis. In addition, it was shown that AF is associated with smaller brain volumes as compared to non-AF patients, and this association was stronger in patients with persistent/permanent forms of AF and with increased time from the diagnosis of the disease (43).

This spectrum of AF-related cerebral phenotypic alterations can be broadly brought back to three possible phenomena: (1) subclinical micro-embolic events; the repetitive recurrence of these events, causing SCIs and WMHs, might contribute in reducing brain volume and affecting cognitive function; (2) oral anticoagulant therapy, particularly in case of warfarin therapy (44), may partly promote cerebral microbleeds; (3) the irregular rhythm may directly impact cerebral circulation, resulting in cerebral lesions (SCIs, microbleeds, WMHs) and atrophy.

Different from the first two mechanisms, the latter has seldom been investigated, most probably due to the evident technical difficulties in assessing the cerebral circle downstream of the Willis circle. Scant evidence from the end of the past century using transcranial Doppler (TCD) already suggested that AF might lower mean regional cerebral perfusion (45), but the scientific community had to wait until 2018 for the seminal work of Gardasdottir (46) for definitive proof. In this work, brain perfusion was estimated with phase contrast MRI in a large cohort of patients from the AGES-Reykjavik Study: individuals with persistent AF showed a reduced mean cerebral blood flow as compared to paroxysmal AF patients (in SR at the time of the MRI; −8%) and controls with no history of AF (−13%). In addition, in a subsequent experiment (47), the same group demonstrated that successful electrical cardioversion in persistent AF individuals was associated, after at least 10 weeks of SR maintenance, with an improvement of brain perfusion and cerebral blood flow measured by both arterial spin labeling (ASL) and phase contrast MRI, while no change in perfusion or blood flow was detected in those individuals where cardioversion was unsuccessful.

In addition to the reduction of the mean cerebral blood flow, AF with its irregular ventricular activation might also have a beat-to-beat impact on cerebrovascular circulation. This hypothesis has been demonstrated *in silico* (48). Based on a cerebral fluid-dynamics setup with two coupled lumped-parameter models (of the systemic and cerebral circulation, respectively), AF is related to transient and repetitive critical cerebral hemodynamic events in the distal cerebral circle, consisting of brief but incessant periods of deep cerebral hypoperfusions or hypertensive events. The repetitive occurrence of these critical events might at least partly explain the genesis of SCIs/WMHs (transient hypoperfusions) and cerebral microbleeds (transient hypertensive events), which could accumulate over time and determine the progressive cerebral damage linked to cognitive decline and dementia.

*In vivo* validation of these computational findings, given the limitations of the widely adopted noninvasive techniques assessing cerebral hemodynamics (TCD and cerebral ALS-MRI) (49, 50) not powered to assess beat-to-beat deep microcirculatory dynamics, is challenging. Our group, for this aim, proposed spatially resolved near-infrared spectroscopy (SRS-NIRS) (51), a noninvasive technique mainly used to monitor cerebral tissue oxygenation in critical care, with the ability to provide a noninvasive assessment of the cerebral microcirculation with high temporal resolution, sensitive to beat-to-beat variations when used with a high sampling frequency (20 Hz). Cerebral SRS-NIRS and noninvasive systemic hemodynamic monitoring were recorded before and after elective electrical cardioversion in 53 AF/atrial flutter (AFL) patients (52), analyzing the total hemoglobin index (THI), a proxy of deep cerebral blood flow. In case of successful SR restoration, in front of a nonsignificant decrease in arterial blood, both hypoperfusive and hyperperfusive/hypertensive microcirculatory events were significantly reduced. These findings represent the first *in vivo* demonstration that SR restoration by ECV significantly improves cerebral microcirculation on a beat-to-beat level.

**FIGURE 3:**
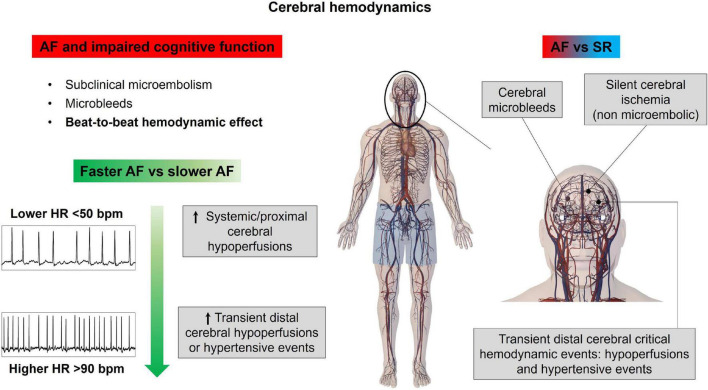
Atrial fibrillation effects on cerebral hemodynamics. AF, atrial fibrillation; HR, heart rate; SR, sinus rhythm.

Additional intriguing insights on the association between AF and cognitive decline/dementia derive from an interesting 10-year follow-up study by Cacciatore (53). In this experience, AF patients presenting low (<50 bpm) compared to high (>90 bpm) median ventricular response were predictive of dementia onset. In fact, as suggested by computational data (54), higher ventricular rates relate to a progressive increase in critical cerebral hemodynamic events (hypoperfusions and hypertensive events) at the distal cerebral circle, while the excessively slow ventricular response is associated with systemic-proximal cerebral (up to the middle cerebral artery) hypoperfusions. Altogether, these findings, despite the results of the aforementioned RACE II trial, suggest that a rate control strategy aiming for a median ventricular response lower than 110 bpm appears more beneficial in terms of cerebral circulation. The main concepts of this section are illustrated in Figure 3.

## Atrial fibrillation and coronary circulation

Patients with ongoing AF may present chest pain, ECG abnormalities, such as ST segment depression, and troponin release, even in the absence of classical obstructive epicardial coronary disease, particularly at high ventricular rates (55–57).

The coronary circulation presents some peculiar characteristics: the blood flow is mainly diastolic and a complex interaction occurs between the driving pressure (aortic pressure) and extravascular forces (intramural and endocavitary pressure) compressing the microvasculature of the different myocardial layers, in particular the subendocardium. In this setting, AF-related hemodynamics may determine blunted coronary flow reserve even in the presence of normal epicardial coronary arteries (55, 58).

The AF patients present downregulation of endothelial nitric oxide synthase (eNOS) (59), accounting for reduced nitric oxide-dependent vasodilation, as well as a potential influence on neurohumoral factors (60) [elevated levels of atrial natriuretic peptide (ANP) and brain natriuretic peptide (BNP), which mediate a shift of the vascular tone toward vasoconstriction], and increased sympathetic tone (61) (which increases coronary vascular resistance, reducing coronary flow reserve in response to increased myocardial energy demand). On top of these mechanisms, a direct detrimental hemodynamic effect may also relate to the irregular RR intervals, similar to the altered cerebral patterns of the microcirculatory perfusion, transient cerebral hypoperfusion, and hypertensive events, observed for the cerebral circulation during AF (48, 52, 54, 62). A computational multiscale (0D-1D) cardiovascular model including left heart mechanics and arterial tree fluid dynamics, together with a one-dimensional description of the epicardial coronary circulation, was designed (63). Based on this heart-arterial-coronary model, AF was simulated at different median ventricular responses (50, 70, 90, 110, 130 bpm), assessing the impact of the arrhythmia at different ventricular rates on the left anterior descending (LAD) flow waveform, as well as on coronary blood flow perfusion. The LAD flow waveform emerged as severely affected by AF, resulting, in particular, in a net decrease of coronary blood flow above 90–110 bpm. In addition, oxygen consumption monotonically increased with the ventricular rate (as estimated by rate pressure product), underlying how exceeding 90–110 bpm likely causes an imbalance in the oxygen supply-demand ratio.

To further study AF-related coronary microcirculation, a similar 1D-0D multiscale model of the entire human cardiovascular system enriched by detailed mathematical modeling not only of the epicardial coronary arteries but also of their downstream microcirculatory districts, subdivided into three layers (subepicardium, midwall, and subendocardium), has been designed (64). In this setting, AF and SR have been simulated at different ventricular rates (75, 100, 125 bpm), and the mean microcirculatory blood flow per beat across the different myocardial layers, in the districts downstream of the three coronary arteries [LAD; left circumflex artery, LCx; and right coronary artery, RCA], was compared. The major findings of this analysis were that the microcirculatory blood flow decreases across all myocardial layers, and at all ventricular rates, in AF compared to SR. In particular, the most affected were the subendocardial layers of the microcirculatory districts downstream of the left coronary arteries (LAD, LCx), where increased left endoventricular pressure during AF plays a role in increasing microvascular resistance of the subendocardial vessels. Of note, there was a more significant reduction of microvascular blood flow across all cardiac layers in AF simulations at the higher ventricular response (100, 125 bpm), as compared to the corresponding SR simulations, supporting the concept that fast ventricular rates during arrhythmia appear detrimental.

In the end, a 1D model of coronary circulation, with an assigned flow rate at the aorta root as an upstream boundary condition and constant pressure as a distal outflow boundary condition, was proposed to study the impact of different kinds of arrhythmias on coronary circulation (65). The authors found that coronary blood flow, defined as the net blood flow through left and right coronary arteries, was significantly affected by arrhythmias. In particular, during bigeminy, trigeminy, and quadrigeminy, coronary blood flow decreased by 28, 19, and 14% with respect to the baseline pacing at rest (60 bpm) and by 33, 22, and 17% with respect to pacing at 160 bpm, respectively.

**FIGURE 4:**
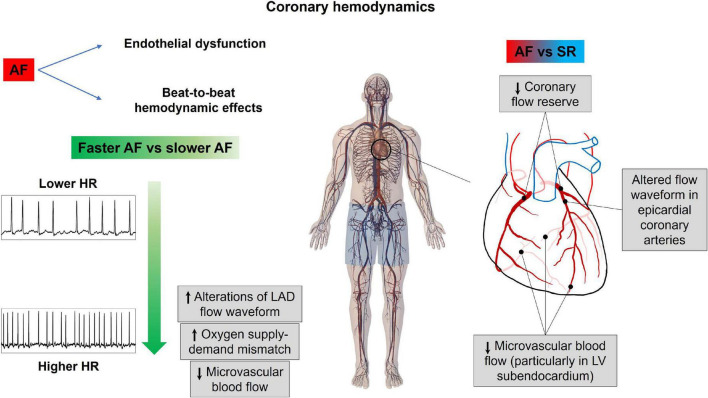
Atrial fibrillation effects on coronary hemodynamics. AF, atrial fibrillation; HR, heart rate; LAD, left anterior descending coronary artery; LV, left ventricle; SR, sinus rhythm.

Overall, these findings are concordant with the available evidence in the literature. Mechanically induced AF diminished coronary flow reserve particularly in the subendocardial layers of dog’s hearts (subendocardial blood flow was reduced by 22%, while subepicardial blood flow was reduced by 9%) (66). Moreover, Kochiadakis elegantly demonstrated in humans (67), with the use of an intracoronary Doppler flow wire, reduced coronary flow reserve in experimentally induced AF compared to right atrial pacing at a similar heart rate, proving the critical role played by the irregular RR interval; interestingly, in the same study, it was also shown that, in case of AF with excessively short RR intervals, the ventricle may not generate enough pressure to open the aortic valve, corresponding to a markedly reduced coronary flow in that specific heartbeat. Figure 4 resumes the main notions regarding how AF affects coronary circulation.

## Limitations

Although computational modeling presents advantages in characterizing the hemodynamic impact exerted by AF, especially in those vascular regions—as the cerebral microcirculation—where the anatomical and structural complexity makes accurate clinical measures difficult to obtain, the following limitations need to be considered. First, the described hemodynamic modeling approaches predict acute hemodynamic effects, not taking into account possible chronic arrhythmia-induced hemodynamic compensatory mechanisms. Second, not all the cardiovascular models presented incorporate beat-to-beat autonomic response, and none of them considers the long-term effects of the autonomic nervous system. Third, the cardiovascular modeling approach is usually calibrated on a generic young healthy subject and validated against available AF hemodynamic literature data. While a number of mechanisms, such as posture and gravity effects, metabolic regulations, and cardiac electromechanics activity, would need to be included to account for different hemodynamic scenarios (from cardiac dysfunctions to astronautical applications), patient-specific cardiovascular modeling surely is emerging as a powerful tool to personalize and integrate cardiac care.

## Conclusion

Growing scientific evidence, mainly based on cardiovascular modeling studies, points toward a relevant impact of AF on different aspects of physiological cardiovascular functioning, ranging from a broad impact on heart mechanics and systemic circulation to more specific influences on key vascular beds such as the cerebral and the coronary circles. Altogether, these data, if considered with the recent results of the EAST-AFNET 4 trial (12), highlight the critical role of sinus rhythm maintenance in improving not only patient’s prognosis but also patient’s hemodynamics, with likely benefit in cognitive function and ischemic symptoms. In addition, in case sinus rhythm could not be maintained over time, a strict rate control in permanent AF patients might be advisable to limit the hemodynamic impact of the arrhythmia.

## Author contributions

MA and AS conceived the review. All authors wrote the article and approved the submitted version.
